# Identification and validation of molecular subtype and prognostic signature for lung adenocarcinoma based on neutrophil extracellular traps

**DOI:** 10.3389/pore.2023.1610899

**Published:** 2023-04-18

**Authors:** Yanhua Zuo, Guangyi Leng, Ping Leng

**Affiliations:** ^1^ Department of Pharmacy, The Affiliated Hospital of Qingdao University, Qingdao, China; ^2^ Laboratory of Drug Metabolism and Pharmacokinetics, Shenyang Pharmaceutical University, Shenyang, China

**Keywords:** immunotherapy, lung adenocarcinoma, tumor microenvironment, prognostic signature, neutrophil extracellular traps

## Abstract

**Background:** Neutrophil Extracellular Traps (NETs) are fibrous networks made of DNA-histone complexes and proteins protruded from activated neutrophils. Accumulating evidences have highlighted the vital role of NETs in tumor progression and diffusion. However, limited systematic studies regarding the role of NETs in LUAD have been performed.

**Methods:** Differentially expressed NETs-related genes and their mutation landscape were identified with TCGA database. Consensus clustering analysis was performed to determine the NETs-related subtypes of LUAD. LASSO algorithm was employed to construct a prognostic signature. Moreover, GSE30219 and GSE31210 were used as independent validation. We also constructed a lncRNA-miRNA-mRNA regulatory axis with several miRNA and lncRNA databases.

**Results:** Consensus clustering identified two NETs-related clusters in LUAD. High NETs score was correlated with a favorable overall survival, abundant immune cell infiltration, and high activity of immune response signal pathways. Six NET-related genes (G0S2, KCNJ15, S100A12, AKT2, CTSG, and HMGB1) with significant prognostic value were screened to develop a prognostic signature. LUAD patients with low-risk had a significantly favorable overall survival both in the training set and validation set. Moreover, NETs-related risk score and clinical stage could act as an independent prognostic factor for LUAD patients. Significant correlation was obtained between risk score and tumor immune microenvironment. We also identified lncRNA BCYRN1/miR-3664-5p/CTSG regulatory axis that may be involved in the progression of LUAD.

**Conclusion:** We developed two molecular subtypes and a prognostic signature for LUAD based on NETs-related genes. This stratification could provide more evidences for estimating the prognosis and immunotherapy of LAUD patients.

## Introduction

Lung cancer is second most common malignancy following by breast cancer, with an estimated 228,820 new cases globally (1). Moreover, lung cancer ranks the leading cause of cancer related deaths globally, claiming about 2 million lives in 2019 (2). Lung cancer has a high aggression and recurrence rate, resulting in a poor prognosis, and the 5-year overall survival of lung cancer is only about 20% (2, 3). Without typical clinical symptoms in early stages, lung cancer patients are usually in the advanced stages when initially diagnosed with disease. Among all cases of lung cancer, lung adenocarcinoma (LUAD) is the most common pathological subtype (4). Despite some mutated genes related to the progression of LUAD, including EGFR and ALK, have been identified, the mechanism of its occurrence and development has not been fully elucidated (5, 6). At present, there is no ideal marker for predicting the prognosis of LUAD patients.

Neutrophils, the most abundant endogenous immune effector cells, are referred as a prognosis biomarker and therapeutic strategy for cancer (7). Neutrophil Extracellular Traps (NETs) are fibrous networks made of DNA-histone complexes and proteins protruded from activated neutrophils (8). NETs contain histone and decondensed DNA chromatin from dying neutrophils, which could respond to specific stimuli by a cell death process named NETosis (9, 10). Accumulating evidences have highlighted the vital role of the disorder and dysregulation of NETosis in many diseases, including rheumatoid arthritis, cardiovascular diseases and cancer (11–13). However, there is no systematic study regarding NETs-related genes in the molecular mechanisms and prognosis of LUAD.

With the development of the second-generation gene sequencing technology, many landmark cancer genomics databases were developed, including the Cancer Genome Atlas (TCGA, https://portal.gdc.cancer.gov/) and Gene Expression Omnibus (GEO, https://www.ncbi.nlm.nih.gov/geo/). More and more prognostic signatures had been constructed by big data mining. Herein, we identified two NETs-related molecular subtypes and a prognostic signature for LUAD by mining database. Our result may provide more evidences about the vital role of NETs in the prognosis of LUAD.

## Materials and methods

### Datasets

The collection of 69 NETs-related genes were conducted from previous studies (Supplementary Table S1) (9, 13, 14). The gene expression profile (Level 3 data, RPKM values), single nucleotide variants (SNV) data and matching clinical data of LUAD patients were downloaded from TCGA database. Two GEO datasets (GSE30219 and GSE31210) were retrieved for validation set. Copy number variation (CNV) data of LUAD were isolated from UCSC Xena (https://xena.ucsc.edu/). The mRNA expression data of TCGA, GSE30219 and GSE31210 were standardized and normalized using “sva” package before analysis.

### Identification of expression and mutation atlas

Using the “limma” and “pheatmap” package, we identified the differentially expressed NETs-related genes in LUAD and “*p* < 0.05 and Log^2^ |(Fold Change)| >2” were set as the threshold. The SNV and CNV atlas of NETs-related genes in LUAD were drawn with “maftools” and “RCircos” package, respectively.

### Consensus clustering

In order to identify NETs-related molecular subtypes in LUAD, we conducted consensus clustering analysis with the ConcensusClusterPlus tool in R. In this analysis, cluster numbers were set from 2 to 6 and replicated process repeated 1,000 times. “Survminer” tool in R was used to generate the survival curve of each cluster of LUAD patients. Cluster map was drawn with “pheatmap” package.

### Functional enrichment analysis and gene set enrichment analysis (GSEA)

After obtaining the differentially expressed genes (DEGs) between low NETs-score cohort and high NETs-score cohort, we employed Gene Ontology (GO) and Kyoto Encyclopedia of Genes and Genomes (KEGG) analyses with “clusterProfiler” package to explore the difference of these two cohorts in signal pathway and biological effects. GSEA was also performed to identify the difference of the signal pathway and biological effects between low NETs-score cohort and high NETs-score cohort with “org.Hs.eg.db,” “clusterProfiler” and “enrichplot” package in R.

### Characterization of immune landscape between two NETs-score cohorts

ESTIMATE algorithm was employed to calculate the immunoscore, stromascore, ESTIMATEScore, tumorPurity of each LUAD case. The difference of these scores between low NETs-score cohort and high NETs-score cohort were evaluated with Student’s t-test with “ggpubr” package. To identify immune characteristics of LUAD cases, expression data were submitted into CIBERSORT (https://cibersort.stanford.edu/) and repeated 1,000 times, which could calculate the relative percentage of 22 immune cell types. The difference of 22 immune cell types between low NETs-score cohort and high NETs-score cohort were analyzed with Student’s t-test and the results were visualized with “vioplot” package. Moreover, we also evaluated the difference in the expression of human leukocyte antigen (HLA)-related genes and immune checkpoints using “reshape2,” “ggplot2” and “ggpubr” packages in R.

### Development of prognostic signature based on NETs-related genes

After identifying the significantly prognostic NETs-related genes with univariate Cox regression analysis, we then performed LASSO cox regression analysis, which could compute the exact coefficient values of each identified association. The risk score of each LUAD case was calculated by a computational equation (sum of coefficient value x gene expression). We then separated LUAD patients into low- and high-risk subgroups with the median riskscore value as the cut-off. The overall survival curve of low- and high-risk subgroups were generated with Kaplan-Meier method. C-index and time ROC analysis were performed with “survminer” package to evaluate the performance of this signature in the prognosis prediction.

### lncRNA-miRNA-mRNA regulatory axis

To identify the NETs-related prognostic signature genes (hub gene) possibly associated with the progression of LUAD, we then employed Wilcox-test to evaluate the expression difference in different TNM stage of LUAD patients. The miRNA targets of hub gene were predicted with miRDB (http://mirdb.org/), miRWalk (http://mirwalk.umm.uni-heidelberg.de/) and Targetscan (https://www.targetscan.org/). LncRNA targets of miRNA were predicted with LncBase (https://carolina.imis.athena-innovation.gr/) and RNAInter (http://www.rna-society.org/).

### Validation of the expression of prognostic signature in LUAD cell lines

Human bronchial epithelial cell lines (HBE) and human lung cancer cell lines (A549, H1299, H1650, HCC827, and PC9) were provided by the Core Facility of Sichuan University west China Hospital (Sichuan, China). These cells were cultured in BEGM™ Bronchial Epithelial Cell Growth Medium (BEGM medium, Lonza, United States) or Roswell Park Memorial Institute 1640 (RPMI1640 medium, Gibco, Waltham, MA, United States). All cell lines were cultured at 37°C in a humidified atmosphere of 5% carbon dioxide (CO_2_). Total RNA of cell lines were extracted using a TRIzol kit (Vazyme, Nanjing, China). RT-q PCR experiments were used to verify the expression of the prognostic signature in LUAD cell lines. The human 18 Svedberg Ribosomal RNA (18S rRNA) was used as the endogenous control for quantifying the expression of selected genes. The relative gene expression levels were calculated by using the comparative 2−DeltaDelta Cycle threshold (2^−ΔΔCT^) method.

### Statistical analysis

All statistical analyses were performed with R software (version 4.2.1). Shapiro-Wilk normality test is used to test the normality distribution of samples. The difference in mRNA levels between two groups with Wilcoxon rank sum test. Kruskal-Wallis Test was performed to evaluate the difference among three groups and Bonferroni method was chosen for the correction of *p*-value. Pearson’s or Spearman’s rank correlation analysis was performed to evaluate the correlations between two continuous variables.

## Results

### Defining of the expression and genetic mutation landscape of NETs-related gene in LUAD

**FIGURE 1:**
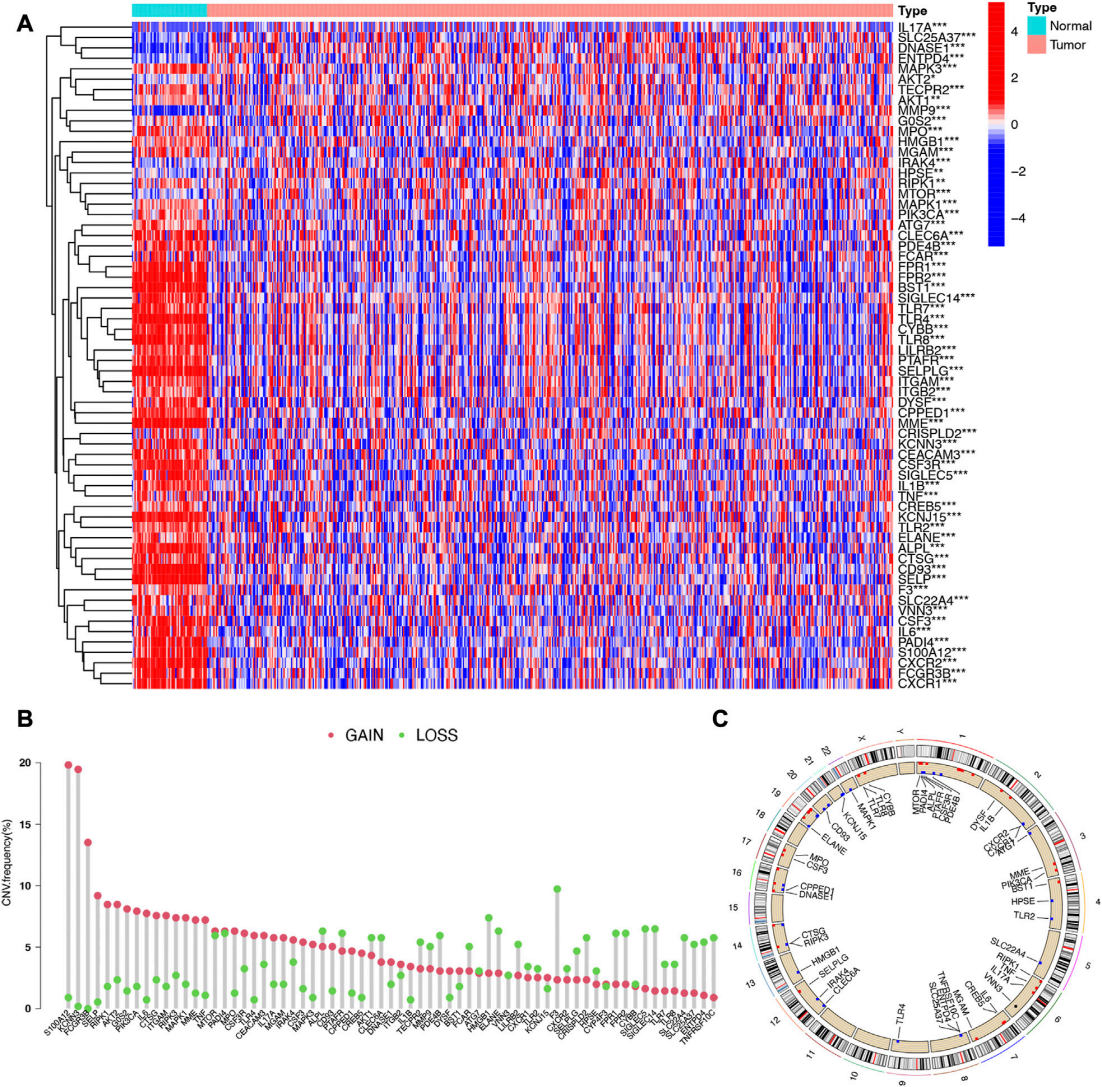
Expression and somatic mutation landscape of NETs-related genes in LUAD. **(A)** Heatmap reveals differentially expressed NETs-related genes in LUAD. **(B,C)** The copy number variation landscape of NETs-related genes in LUAD and their location on chromosomes.

Heatmap of Figure 1A showed differentially expressed NETs-related genes in LUAD. Among 69 NETs-related genes, a total of 64 genes were differentially expressed in LUAD versus normal lung tissues, including 11 upregulated genes and 53 downregulated genes (Figure 1A, Supplementary Table S2). CNV atlas in Figure 1B revealed that half of NETs-related genes had copy number (CNV) amplification while another half of NETs-related genes had a widespread CNV deletion. The location of CNV alteration of NETs-related genes on chromosomes were showed in Figure 1C. Supplementary Figure S1A showed the SNV landscape of NETs-related genes in LUAD, which revealed that TLR4 has a highest frequency of SNV, followed by MGAM and DYSF. We then constructed a protein-protein interaction (PPI) network to further clarify the connections between these NETs-related genes (Supplementary Figure S1B).

### Consensus clustering identified two subtypes in LUAD based on NETs-related genes

**FIGURE 2:**
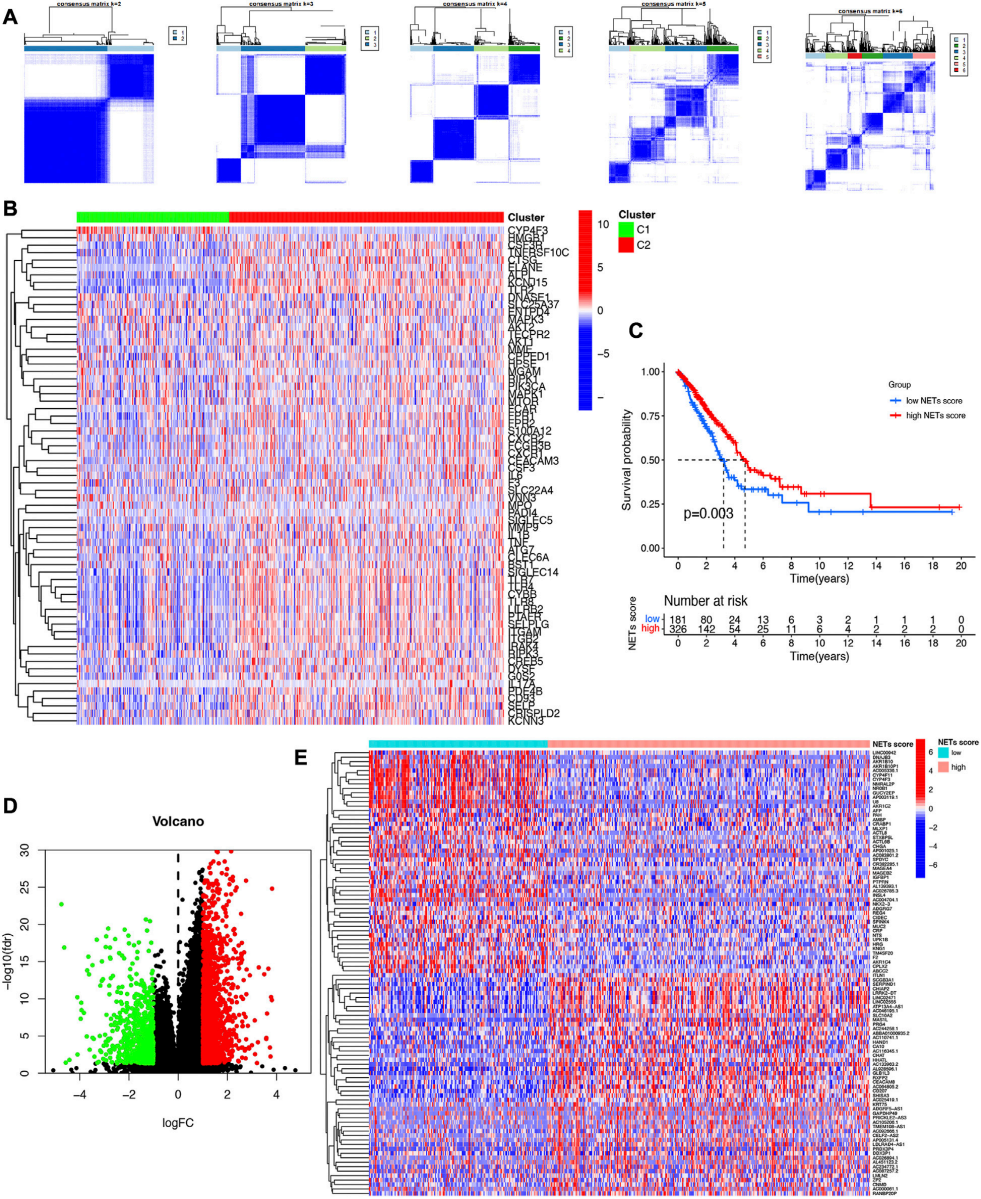
Identification of NETs-related clusters by consensus clustering. **(A)** Consensus clustering matrix about the number of clusters of LUAD. **(B)** Heatmap reveals NETs-related gene expression pattern in different NETs score cluster. **(C)** Overall survival curve of different NETs score cluster. **(D,E)** Volcano plot and heatmap reveal the differentially expressed genes between high NETs score and low NETs score subtypes.

We next determined the numbers of subtype of LUAD based on NETs-related genes using consensus clustering. As a result, two subtypes of LUAD were identified with distinct the genes expression patterns of NETs-related genes after k-means (Figure 2A). Subtype C2 showed overall high NETs-related genes expression pattern was defined as a high NETs score cluster, while Subtype C1 presented low expression pattern was defined as a low NETs score cluster (Figure 2B). Further prognostic analysis revealed that LUAD patients with high NETs score presented a dismal clinical outcome while low NETs score was correlated with a favorable overall survival in LUAD (Figure 2C).

### Identification of signal pathways and biological effects in different NETs score cluster

**FIGURE 3:**
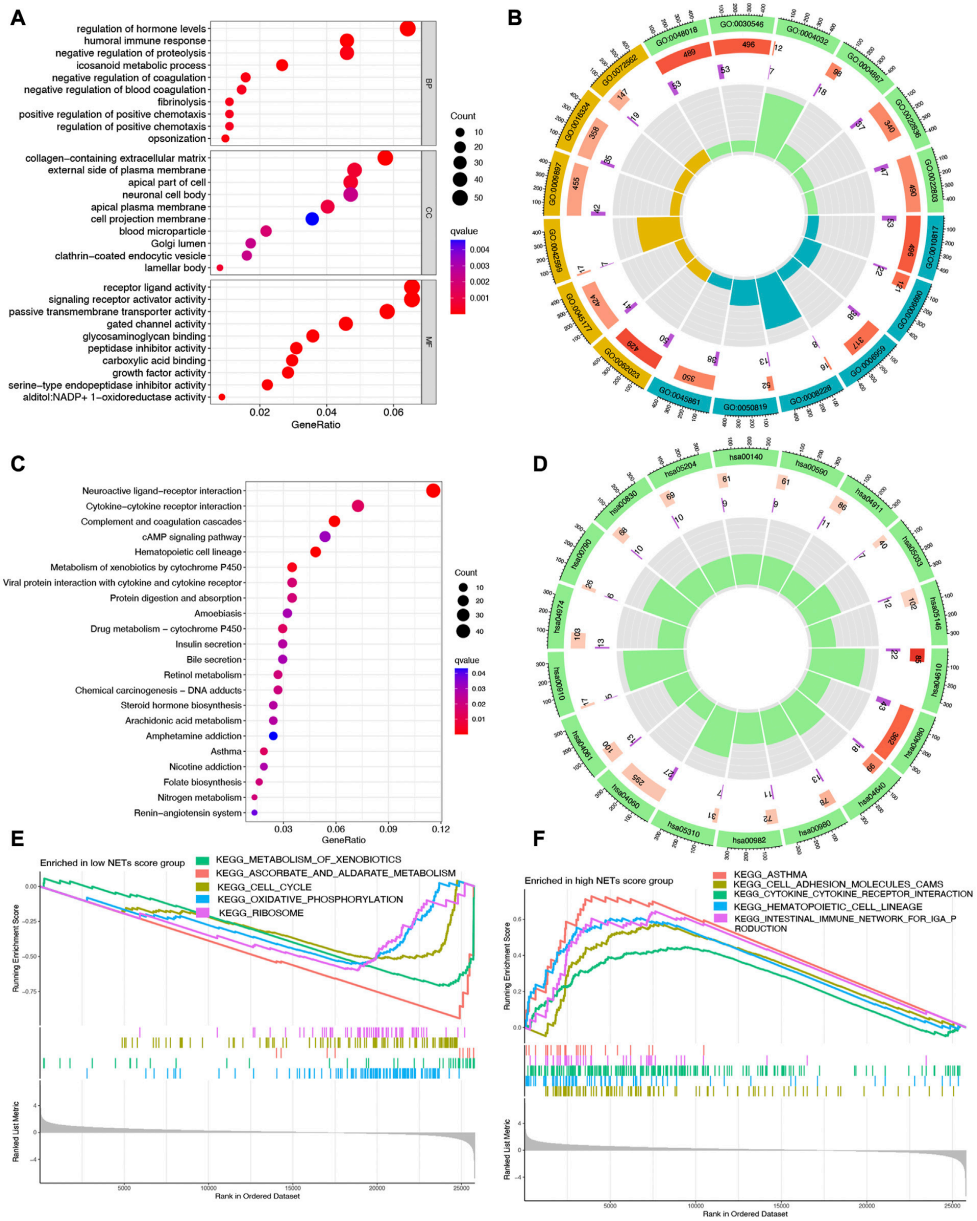
Functional enrichment and GSEA analysis in different cluster. **(A,B)** The enriched items in GO analysis. **(C,D)** The enriched items in KEGG analysis. **(E,F)** The enriched items in low and high NETs score clusters based on GSEA analysis.

Since significantly different clinical outcome were obtained in different NETs score cluster, we then explore the key DEGs, signal pathways and biological effects in each cluster, which may clarify molecular mechanism modulating patients’ prognosis. As a result, a total of 2,120 DEGs were obtained between two different NETs score clusters (Figure 2D). Among these DEGs, the top 50 upregulated genes in two different NETs score clusters were showed in Figure 2E. Interestingly, the result of GO and KEGG pathways revealed that the dysregulated genes of NETs score cluster were mainly correlated with in immunity-related activities, including humoral immune response, regulation of positive chemotaxis, cytokine and cytokine receptor interaction, asthma, complement and coagulation cascades (Figures 3A–D). These data demonstrated a significant correlation between high NETs score cluster and immune active microenvironment. In order to further confirm the difference of signal pathways between high and low NETs score cluster, we performed GSEA analysis. Here, gene sets enriched in low NETs score cluster were correlated with oxidative phosphorylation, metabolism of xenobiotica by cytochrome, cell cycle and ribosome (Figure 3E). On the contrary, gene sets enriched in high NETs score cluster were correlated with cytokine and cytokine receptor interaction, cell adhesion molecules cams, asthma, and intestinal immune network for IgA production (Figure 3F).

### Somatic mutations and tumor microenvironment landscape in different NETs score cluster

**FIGURE 4:**
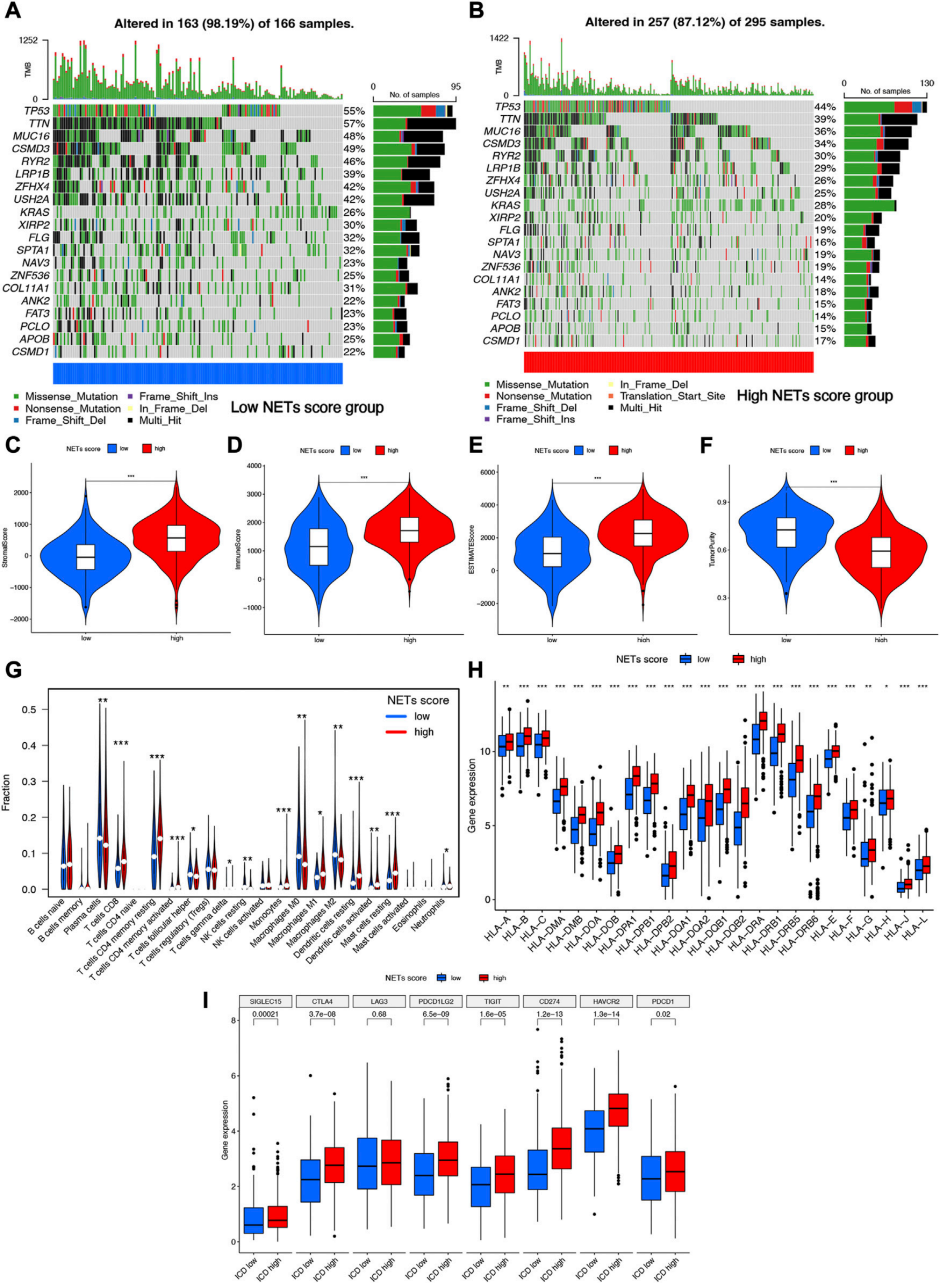
Somatic mutations and tumor microenvironment landscape in different cluster. **(A,B)** The somatic mutation profiles in high and low NETs score cluster. **(C–F)** High NETs score cluster had a higher stromal score, immune score, ESTIMAE score and tumor purity than low NETs score cluster. **(G–I)** The level of immune cells, human leukocyte antigen genes and common immune checkpoints in high and low NETs score cluster.

The somatic mutation profiles in high and low NETs score cluster were summarized in Figures 4A, B. Among them, TP53 (55% vs. 44%), TTN (57% vs. 39%), MUC16 (48% vs. 36%), GSMD3(49% vs. 34%), and RYR2 (39% vs. 30%) ranked the top fine the most frequency gens, exerting a greater somatic mutation frequency in low NETs score cluster than in the high NETs score cluster (Figures 4A, B). Accumulating evidences revealed that NETs played a vital role in innate immune response (12). The current study also clarified analyzed the composition of tumor microenvironment in different NETs score clusters. Overall, high NETs score cluster had a higher stromal score (Figure 4C), immune score (Figure 4D), ESTIMAE score (Figure 4E) and lower tumor purity (Figure 4F) than low NETs score cluster (all *p* < 0.001). The relative percentage of 22 immune cell types in each TCGA LUAD sample was summarized in Supplementary Figure S2A. And the correlation heatmap of each immune cell type was shown in Supplementary Figure S2B. To be more specific, LUAD patients with low NETs score cluster had a higher abundance of plasma cell, follicular helper T cell, NK resting cell, macrophage M0, macrophage M2. However, low NETs score cluster had a lower abundance of CD8 T cell, activated CD4 T cell, memory resting CD4 T cell, activated NK cell, Monocyte, macrophage M1, resting dendritic cell, active dendritic cell, resting mast cell and neutrophils (Figure 4G). Further analysis revealed that the expression of most of the human leukocyte antigen (HLA) genes and common immune checkpoints were higher in LUAD patients with high NETs score cluster (Figures 4H, I).

### Construction and validation of prognostic signature based on NET-related genes

**FIGURE 5:**
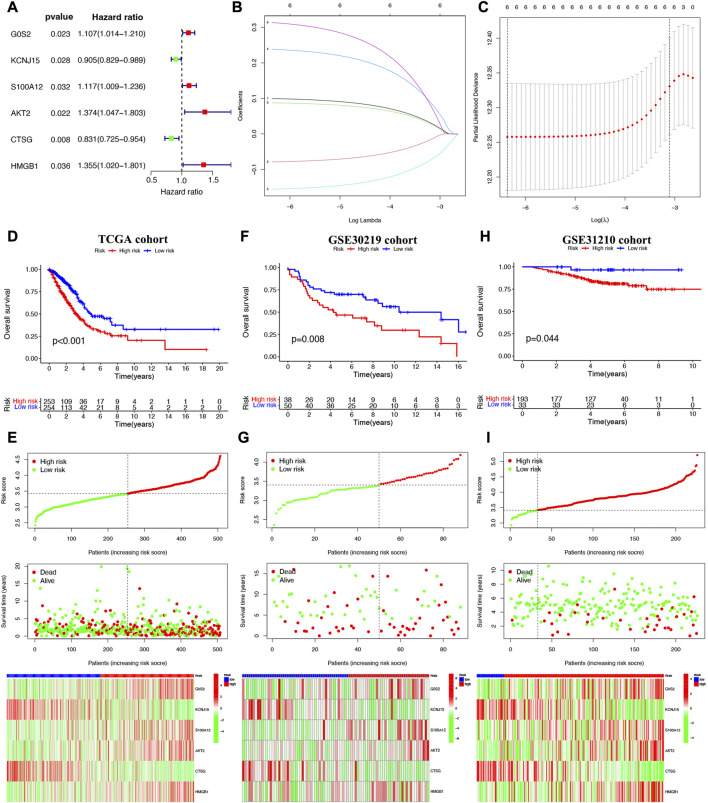
Construction of a NETs-related prognostic signature. **(A)** NETs-related genes with significant prognostic value based on univariate cox regression analysis. **(B,C)** The coefficient and partial likelihood deviance of prognostic signature. Overall survival curve, risk scores distribution, patients’ survival status, gene expression heatmap of NETs-related prognostic signature in TCGA cohort **(D,E)**, GSE30219 cohort **(F,G)** and GSE31210 cohort **(H,I)**.

**FIGURE 6:**
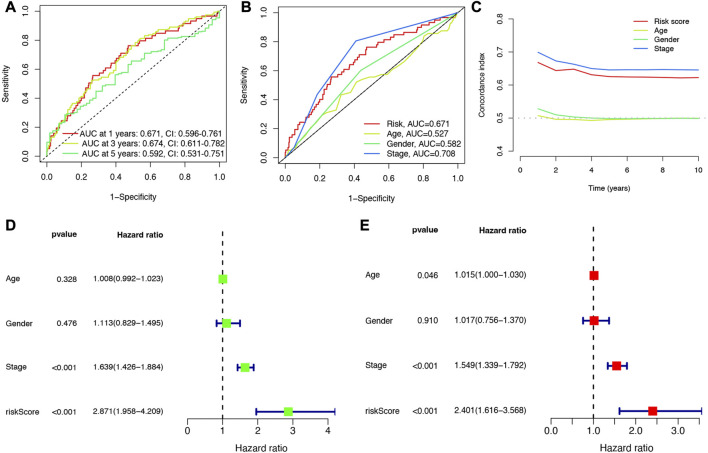
Evaluation of the performance of NETs-related prognostic signature. **(A)** The AUCs under ROC curves for 1-, 3-, and 5-year overall survival. **(B,C)** ROC and C-index compared the diagnosed performance of risk scores with clinical characters. **(D,E)** Univariate and multivariate cox regression considering clinical parameters and NETs-risk score.

Cox univariate analysis indicated that 6 NET-related genes (G0S2, KCNJ15, S100A12, AKT2, CTSG, and HMGB1) were significantly correlated with the prognosis of LUAD patients (Figure 5A). These 6 genes were submitted for LASSO regression analysis and, as a result, all of them were selected in the prediction model (Figures 5B, C). The risk score of LUAD cases were calculated with the algorithm below: Risk score = (0.100)*G0S2 + (−0.079)*KCNJ15 + (0.088)*S100A12 + (0.238)AKT2 + (−0.156)*CTSG + (0.314)*HMGB1. We further investigated the correlation between clinical outcome and risk score. As expected, LUAD patients with high risk score were associated with a poor overall survival in TCGA cohort (Figure 5D, *p* < 0.001). The number of dead statuses in high risk group was more than that in low risk group (Figure 5E). We also obtained the similar results in GSE30219 cohort (Figures 5F, G) and GSE31210 cohort (Figures 5H, I). Further ROC curve indicated that the AUC value in 1-year, 3-year, and 5-year overall survival were 0.671, 0.674, and 0.592, respectively (Figure 6A). Diagnostic ROC curve and C-index revealed that risk score and clinal stage was relatively good predictors for the clinical outcome of LUAD patients (Figures 6B, C). Moreover, univariate and multivariate analysis revealed that NETs-related risk score and clinical stage could act independent prognostic factors for LUAD patients (Figures 6D, E).

### NETs-risk score was significantly correlated with immune cell infiltration in LUAD

**FIGURE 7:**
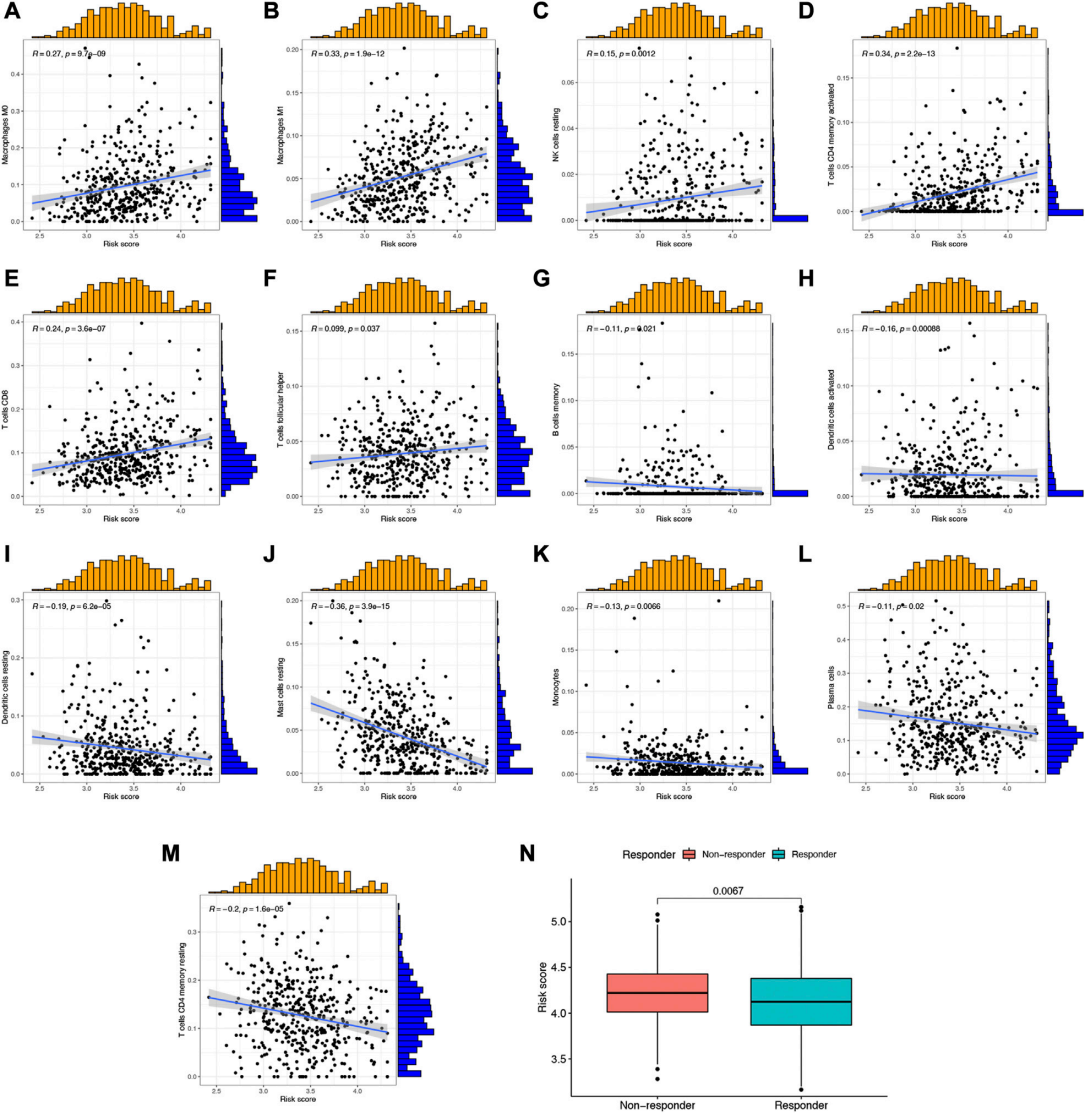
The correlation between NETs-related risk score and immune cell level as well as TIDE score. **(A–M)** Scatter plots revealed the correlation of risk score and the abundance of different immune cells. **(N)** The risk score in immunotherapy response and immunotherapy non-response group.

Since NETs played a vital role in the progression of cancer and immunological responses, it was necessary to clarify the correlation between NETs-risk score and tumor microenvironment. As shown in Figures 7A–F, the abundance of Macrophages M0, Macrophages M1, NK cells resting, T cells CD4 memory activated, T cells CD8, and T cells follicular helper was positively correlated with NETs-risk score in LUAD. As NETs-risk score increased, the abundance of B cells memory, Dendritic cells activated, Dendritic cells resting, Mast cells resting, Monocytes, Plasma cells, and T cells CD4 memory resting decreased (Figures 7G–M). Moreover, TIDE was used to assess the predictive value of NETs-risk score in the potential clinical efficacy of immunotherapy. Interestingly, immunotherapy non-response group had a higher risk score than that in immunotherapy response group (Figure 7N, *p* = 0.0067), demonstrating that LUAD patients with low NETs-risk score would be more possibly benefited more from immunotherapy.

### Construction of lncRNA-miRNA-mRNA regulatory axis

**FIGURE 8:**
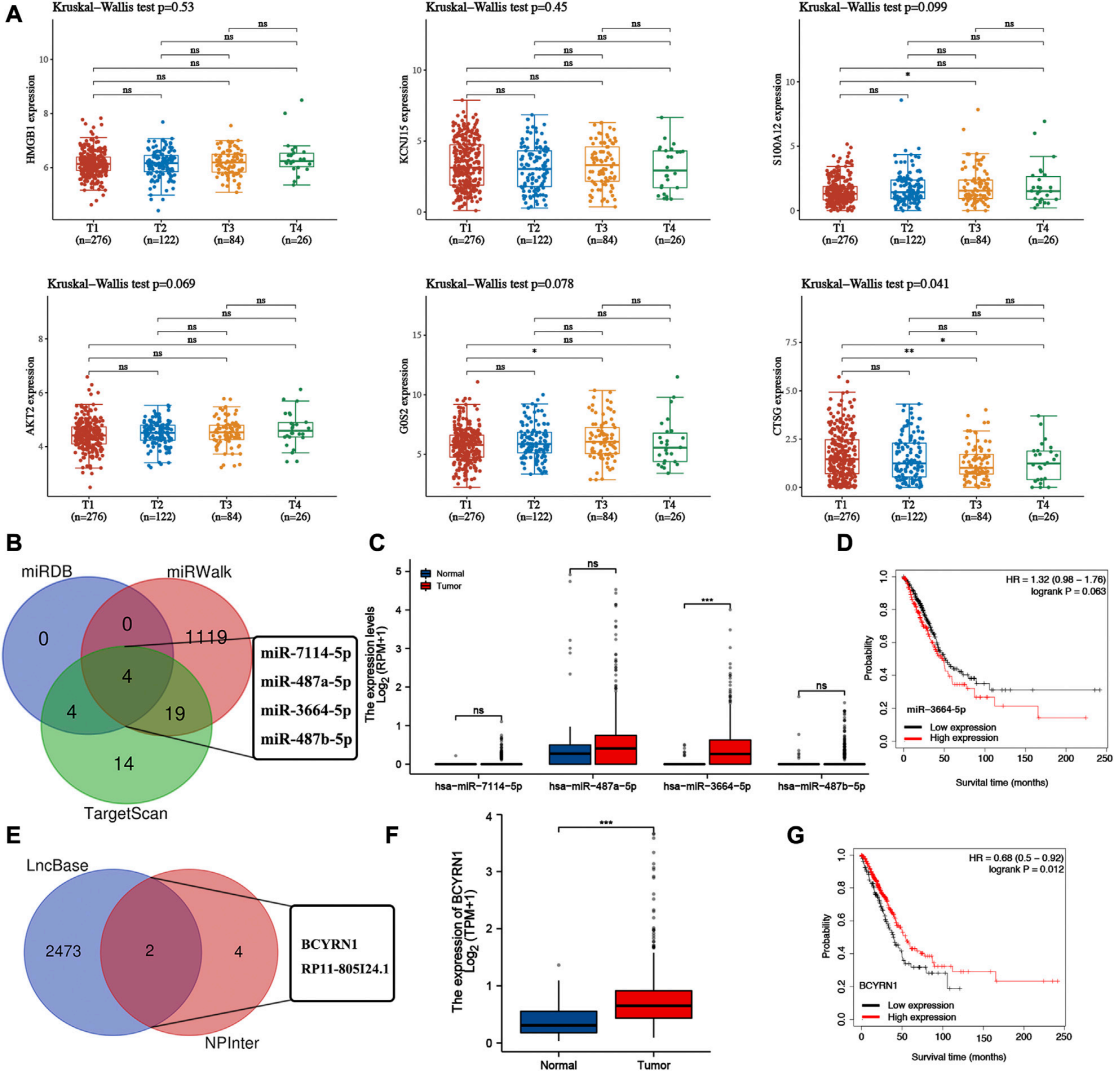
Construction of lncRNA-miRNA-mRNA regulatory axis. **(A)** The expression of NETs-related prognostic signature genes in different clinical stage group of LUAD patients. **(B)** miRNA targets of CTSG predicted by miRDB, miRWalk and TargetScan. **(C,D)** The expression and prognostic value of miRNA targets in LUAD. **(E)** LncRNA targets of miR-3664-5p predicted by lncBase and RNAIter. **(F,G)** The expression and prognostic value of lncRNA targets in LUAD. *p < 0.05, **p < 0.01, ***p < 0.001.

The expression of G0S2, HMGB1, S100A12, AKT2 were upregulated (Supplementary Figures S3A–D) while the expression of CTSG and KCNJ15 were decreased in most of LUAD cell lines compared with human bronchial epithelioid cells (HBE) (Supplementary Figures S3E, F). We then analyzed the correlation between the expression of NETs-prognostic signature genes and clinical stage. Here, only CTSG expression was significantly correlated clinical stage in LUAD (Figure 8A). Thus, we selected CTSG for further analysis. In order to further clarified the mechanism of CTSG in the progression of LUAD, we then constructed a lncRNA-miRNA-mRNA regulatory axis. Using miRDB, miRWalk and TargetScan, we identified miR-7114-5p, miR-487a-5p, miR-3664-5p and miR-487b-5p as the miRNA targets of CTSG (Figure 8B). Among these four miRNA targets, only miR-3664-5p was significantly dysregulated in LUAD (Figure 8C, *p* < 0.001) and correlated with clinical outcome though the *p*-value was 0.063 (Figure 8D). Thus, miR-3664-5p was the most promising miRNA target of CTSG. We then used RNAInter and lncBase to further explore its upstream lncRNA targets. As shown in Figure 8E, lncRNA BCYRN1 and RP11-805I24.1 were suggested as the targets of miR-3664-5p. However, only BCYRN1 was significantly dysregulated in LUAD (Figure 8F, *p* < 0.001) and correlated with clinical outcome in LUAD (Figure 8G), indicating BCYRN1 as the most promising lncRNA target. All in all, we identified lncRNA BCYRN1/miR-3664-5p/CTSG regulatory axis in the progression in LUAD. Further study would be performed to confirm the results.

## Discussion

NETs are decondensed extracellular chromatin filaments and play a vital role in innate immune response (15). Accumulating evidences have shown that NETs are involved in many biological processes of cancer, including invasion and evasion (16). Moreover, NETs could also catch circulating cancer cells and facilitate metastasis (12). Interestingly, NETs could serve as therapeutic target and associate with clinical outcome in cancer (9, 17). In the current study, we performed a systematic study regarding NETs-related genes in the molecular mechanisms and prognosis of LUAD.

Based on the expression pattern of NETs-related genes, we conducted consensus clustering analysis and two NETs-related clusters of LUAD were obtained. High NETs score cluster was correlated with a favorable overall survival rate, abundant immune cell infiltration, high immuneScore, high sromaScore and high ESTIMATEScore, and high activity of immune response signal pathways, referring to “hot tumor” (18). Further analysis revealed that high NETs score cluster was correlated with active immune activities, including cytokine and cytokine receptor interaction, cell adhesion molecules cams, asthma, and intestinal immune network for IgA production. “Hot tumors” with significant T-cell infiltration are linked to better immune therapy efficacy (19). Cancer patients with “Hot tumors” phenotype was associated with favorable clinical outcome (20). Actually, previous study also demonstrated that LUAD patients with high immuneScore, sromaScore and ESTIMATEScore were associated with better clinical outcome (18). Low NETs score cluster had a higher abundance of plasma cell, follicular helper T cell, NK resting cell, macrophage M0, macrophage M2. However, high NETs score indicated a lower abundance of CD8 T cell, activated CD4 T cell, memory resting CD4 T cell, activated NK cell, Monocyte, macrophage M1, resting dendritic cell, active dendritic cell, resting mast cell and neutrophils. Higher level of CD8 T cell, activated CD4 T cell, memory resting CD4 T cell, activated NK cell and macrophage M1 were associated with better immune response. While macrophage M2 could promote the progression of many types of cancer, including LUAD (21–23). Without immune checkpoints expression, those patients would not benefit from immunotherapy. And the patients with high immune checkpoints expression would benefit from immunotherapy such as anti-PD1 or anti-CTLA4 drug. Tumor cell HLA expression helps antitumor immune response (24). This may be one of the reasons why LUAD patients in High NETs score cluster was correlated with the favorable overall survival.

Six NET-related genes (G0S2, KCNJ15, S100A12, AKT2, CTSG and HMGB1) with significant prognostic value were screened to develop a prognostic signature. We found that LUAD patients with low-risk score had a favorable overall survival rate both in the training set and validation set. Moreover, NETs-related risk score and clinical stage could act as independent prognostic factors for LUAD patients. These data suggested that this NETs-related prognostic signature had a good performance in the prognosis prediction of LUAD patients. As far as we known, only two NETs-related prognostic signatures had been constructed in cancers. Chen et al. constructed a lncRNA signature based on NETs-related gene in non-small-cell lung cancer, which had good performance in predicting survival (10). Another bioinformatics analysis also developed a NETs-related prognostic signature for pan-cancer (9).

Some of six NET-related prognostic biomarkers (G0S2, KCNJ15, S100A12, AKT2, CTSG and HMGB1) also played a vital role in LUAD. High AKT2 drives cancer progression in lung adenocarcinoma (25). Moreover, silencing of AKT2 could reduce cellular motility and invasion in LUAD (26). Another study revealed that high HMGB1 may induce tumorigenesis, metastasis and chemotherapy resistance in lung cancer (27).

We also identified lncRNA BCYRN1/miR-3664-5p/CTSG regulatory axis that may be involved in the progression of LUAD. BCYRN1 was suggested as an oncogenic lncRNA in diverse cancers (28). Moreover, BCYRN1 could accelerate glycolysis and tumor progression *via* miR-149/PKM2 axis in lung cancer (29). Moreover, BCYRN1 could also regulate cell metastasis in lung cancer (30). miR-3664-5p also exert vital functions in certain types of cancer. miR-3664-5P could inhibit the proliferation and metastasis of gastric cancer *via* NF-κB signaling pathway (31). CTSG was suggested as a prognostic biomarker in certain types of cancer including bladder cancer, LUAD, oral squamous cell carcinoma (32–34). In our further study, we would focus on the verification of the vital of BCYRN1/miR-3664-5p/CTSG regulatory axis in the progression of LUAD *via in vitro* and *in vivo* analysis.

There are some limitations of our study. The 5-year survival in high and low risk score of TCGA cohort was only about 10% difference and the clinical relevance is questionable. It would be better to verify our prognostic signature using another dataset. Moreover, the expression and prognostic role of prognostic signature genes should be verified using clinical tissues.

## Conclusion

In conclusion, we identified two new molecular subtypes and a prognostic signature for lung adenocarcinoma based on NETs-related genes. This stratification could provide more evidence for estimating prognosis and immunotherapy of LAUD patients.

## Data Availability

The original contributions presented in the study are included in the article/Supplementary Material, further inquiries can be directed to the corresponding author.

## Supplementary material

SUPPLEMENTARY FIGURE S1 The mutation landscape and interaction network of NETs-related gens. **(A)** Oncoprint visualization of the mutation of NETs-related gens in LUAD. **(B)** Protein-protein interactions among NETs-related gens.

SUPPLEMENTARY FIGURE S2 Immune landscape of high and low NETs score cluster. **(A)** Relative proportion of immune infiltration in high and low NETs score cluster. **(B)** Correlation heatmap among 22 immune cell types.

SUPPLEMENTARY FIGURE S3 The expression of prognostic signature in LUAD cell lines. The expression of G0S2 , **(A)** HMGB1 **(B)**, S100A12 **(C)**, AKT2 **(D)**, CTSG **(E)** and KCNJ15 **(F)** in LUAD cell lines and human bronchial epithelioid cells (HBE).
