# Supplementary figures and images for: Identification and validation of molecular subtype and prognostic signature for lung adenocarcinoma based on neutrophil extracellular traps
Source: Pathol Oncol Res. 2023 Apr 18;29:1610899. doi: 10.3389/pore.2023.1610899 (PMC10151567; doi:10.3389/pore.2023.1610899)

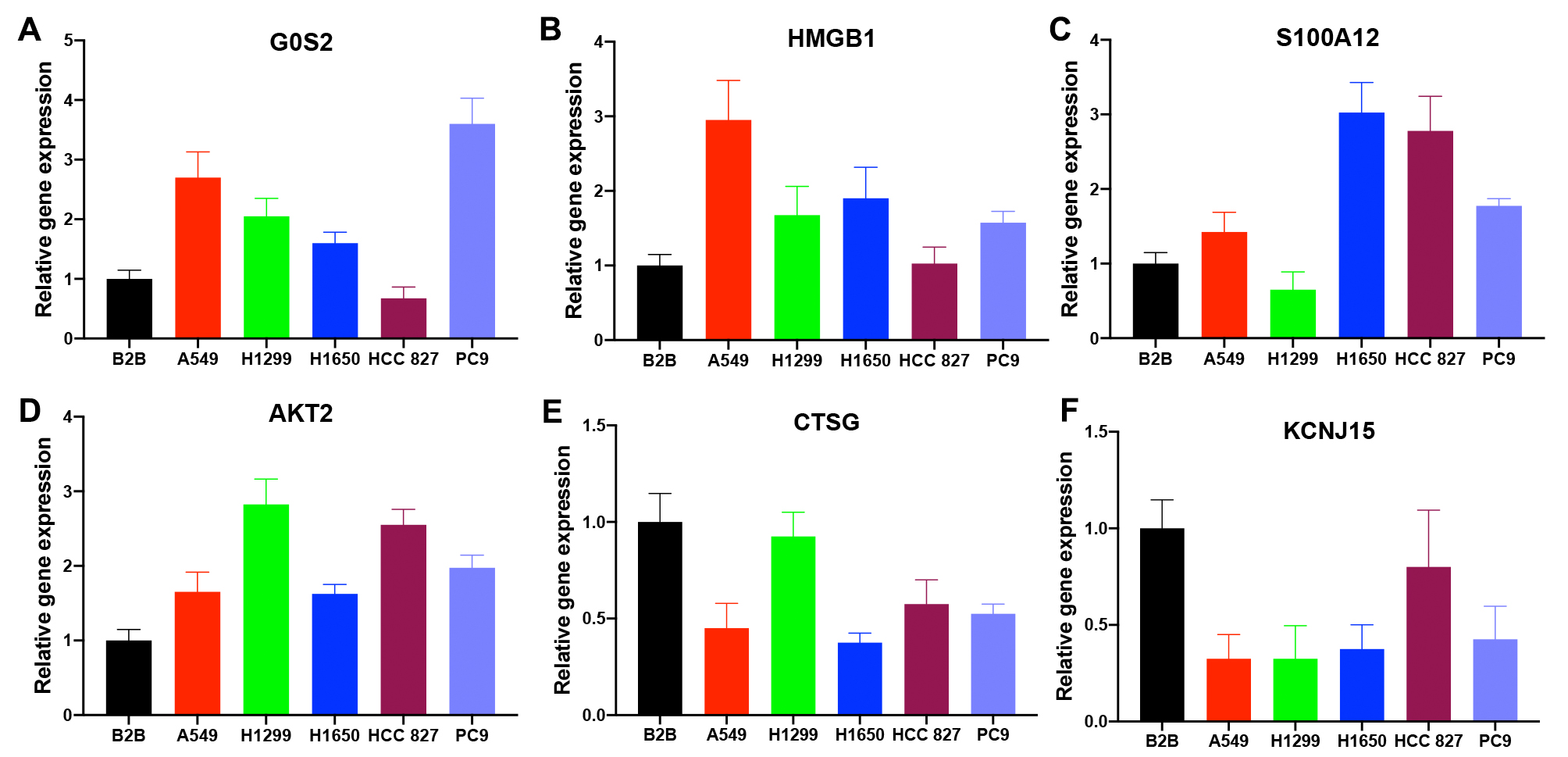

Supplement: Supplementary file 1 [file Image3.JPEG]

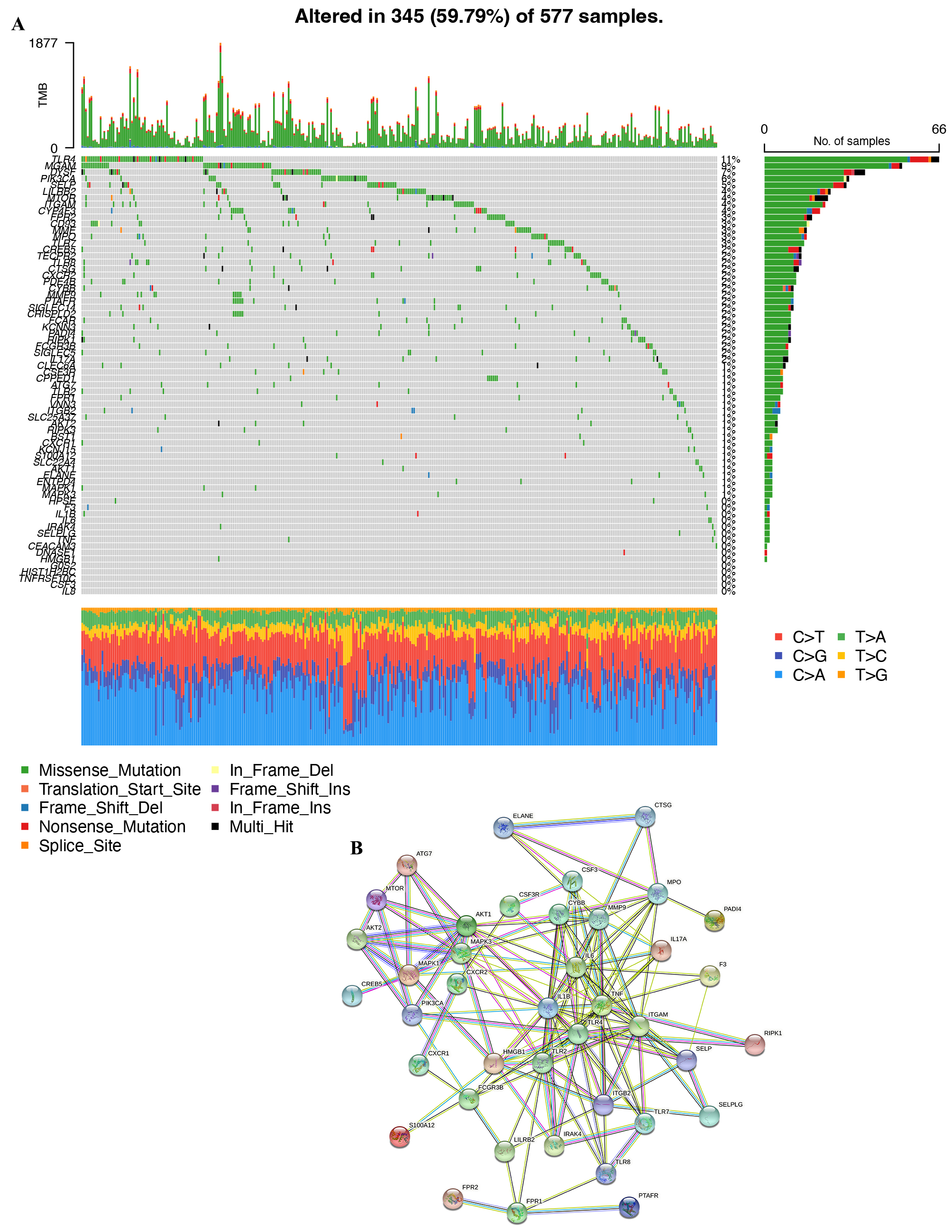

Supplement: Supplementary file 3 [file Image1.JPEG]

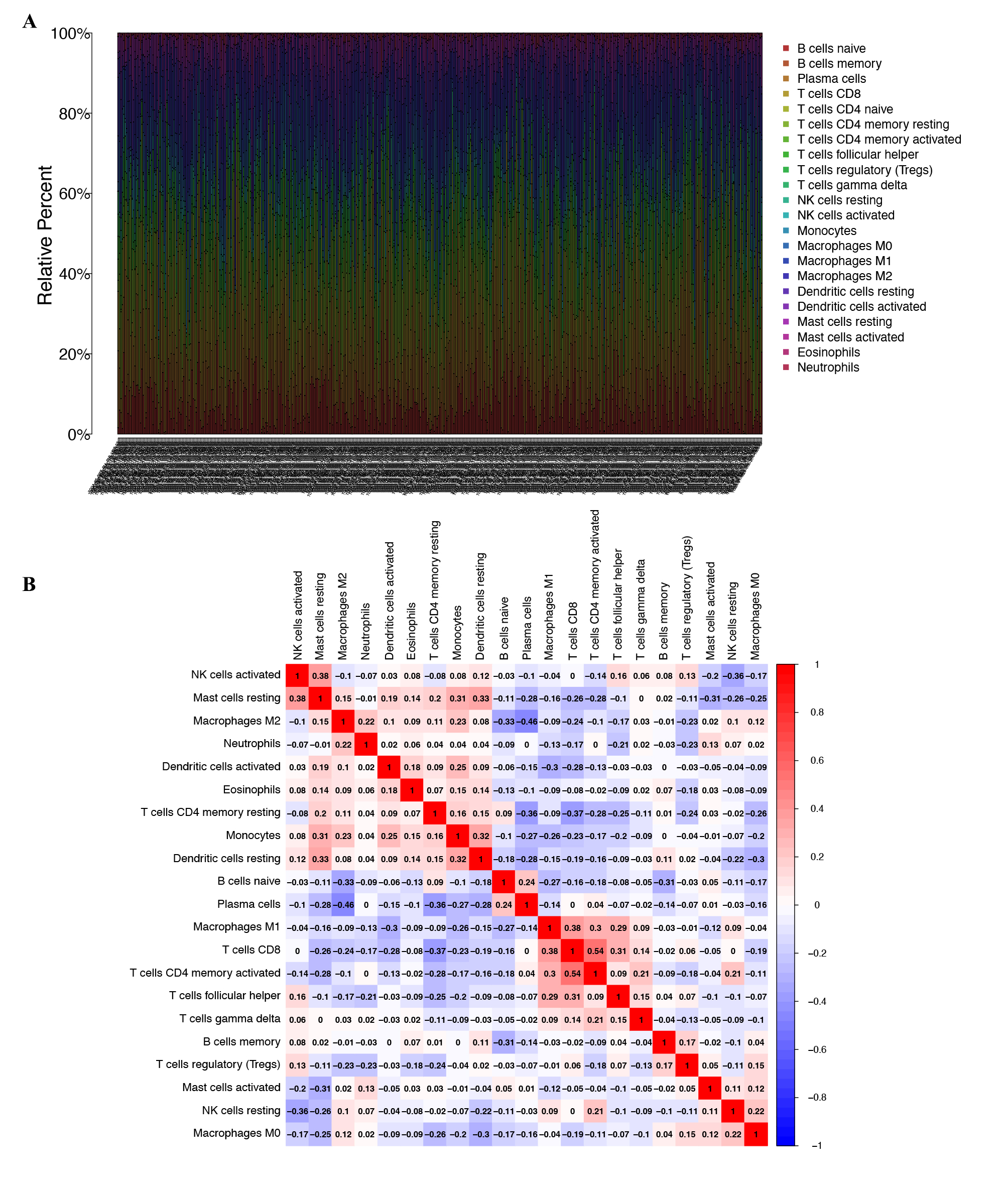

Supplement: Supplementary file 4 [file Image2.JPEG]
