# Supplementary material for: Identification and validation of molecular subtype and prognostic signature for lung adenocarcinoma based on neutrophil extracellular traps
Source: Pathol Oncol Res. 2023 Apr 18;29:1610899. doi: 10.3389/pore.2023.1610899 (PMC10151567; doi:10.3389/pore.2023.1610899)
Supplement: Supplementary file 2 [file Table1.DOCX]

**Supplementary table 1. Neutrophil extracellular traps-related genes obtained from previous study**

| HGNC symbol | Entrez Gene | Ensembl | Description (coding protein) |
| --- | --- | --- | --- |
| ALPL | 249 | ENSG00000162551 | Alkaline phosphatase, biomineralization associated |
| BST1 | 683 | ENSG00000109743 | Bone marrow stromal cell antigen 1 |
| CD93 | 22918 | ENSG00000125810 | CD93 antigen |
| CEACAM3 | 1084 | ENSG00000170956 | Carcinoembryonic antigen-related cell adhesion molecule 3 |
| CREB5 | 9586 | ENSG00000146592 | Cyclic AMP-responsive element-binding protein 5 |
| CRISPLD2 | 83716 | ENSG00000103196 | Cysteine rich secretory protein LCCL domain containing |
| CSF3R | 1441 | ENSG00000119535 | Colony stimulating factor 3 receptor |
| CYP4F3 | 4051 | ENSG00000186529 | Cytochrome P450 family 4 subfamily F member 3 |
| DYSF | 8291 | ENSG00000135636 | Dysferlin |
| FCAR | 2204 | ENSG00000186431 | Fc fragment of IgA receptor |
| FCGR3B | 2215 | ENSG00000162747 | Fc fragment of IgG receptor IIIb |
| CPPED1 | 55313 | ENSG00000103381 | Calcineurin like phosphoesterase domain containing |
| FPR1 | 2357 | ENSG00000171051 | Formyl peptide receptor 1 |
| FPR2 | 2358 | ENSG00000171049 | Formyl peptide receptor 2 |
| G0S2 | 50486 | ENSG00000123689 | G0/G1 switch 2 |
| HIST1H2BC | 8347 | ENSG00000180596 | H2B clustered histone 4 |
| HPSE | 10855 | ENSG00000173083 | Heparanase |
| CXCR1 | 3577 | ENSG00000163464 | C-X-C motif chemokine receptor 1 |
| CXCR2 | 3579 | ENSG00000180871 | C-X-C motif chemokine receptor 2 |
| KCNJ15 | 3772 | ENSG00000157551 | Potassium inwardly rectifying channel subfamily J member 15 |
| LILRB2 | 10288 | ENSG00000131042 | Leukocyte immunoglobulin like receptor B2 |
| MGAM | 8972 | ENSG00000257335 | Maltase-glucoamylase |
| MME | 4311 | ENSG00000196549 | Membrane metalloendopeptidase |
| PDE4B | 5142 | ENSG00000184588 | Phosphodiesterase 4B |
| S100A12 | 6283 | ENSG00000163221 | S100 calcium binding protein 2 |
| SIGLEC5 | 8778 | ENSG00000105501 | Sialic acid binding Ig like lectin 5 |
| SLC22A4 | 6583 | ENSG00000197208 | Solute carrier family 22 member 4 |
| SLC25A37 | 51312 | ENSG00000147454 | Solute carrier family 25 member 37 |
| TECPR2 | 9895 | ENSG00000196663 | Tectonin beta-propeller repeat containing 2 |
| TNFRSF10C | 8794 | ENSG00000173535 | TNF receptor superfamily member 10c |
| VNN3 | 55350 | ENSG0000093134 | Vascular non-inflammatory molecule 3 |
| AKT1 | 207 | ENSG00000142208 | AKT serine/threonine kinase 1 |
| AKT2 | 208 | ENSG00000105221 | AKT serine/threonine kinase 2 |
| ATG7 | 10533 | ENSG00000197548 | Autophagy related 7 |
| CLEC6A | 93978 | ENSG00000205846 | Dectin-2 |
| CSF3 | 1440 | ENSG00000108342 | Granulocyte colony stimulating factor |
| CTSG | 1511 | ENSG00000100448 | Cathepsin G |
| CYBB | 1536 | ENSG00000165168 | NADPH oxidase |
| DNASE1 | 1773 | ENSG00000213938 | Deoxyribonuclease I |
| ELANE | 1991 | ENSG00000197561 | Neutrophil elastase |
| ENTPD4 | 14573 | ENSG00000197217 | Ectonucleoside Triphosphate Diphosphohydrolase 4 |
| F3 | 2152 | ENSG00000117525 | Coagulation Factor III, tissue factor |
| HMGB1 | 3146 | ENSG00000189403 | High mobility group box 1 |
| IL17A | 3605 | ENSG00000112115 | Interleukin 17 |
| IL1B | 3553 | ENSG00000125538 | Interleukin 1 beta |
| IL6 | 3569 | ENSG00000136244 | Interleukin 6 |
| IL8 | 3576 | ENSG00000169429 | Interleukin 8 |
| IRAK4 | 51135 | ENSG00000198001 | Interleukin 1 receptor associated kinase 4 |
| ITGAM | 3684 | ENSG00000169896 | Complement component 3 receptor 3 subunit |
| ITGB2 | 3689 | ENSG00000160255 | Complement component 3 receptor 3 and 4 subunit |
| KCNN3 | 3782 | ENSG00000143603 | Potassium channel, calcium activated |
| MAPK1 | 5594 | ENSG00000100030 | Mitogen-activated protein kinase 1 |
| MAPK3 | 5595 | ENSG00000102882 | Mitogen-activated protein kinase 3 |
| MMP9 | 4218 | ENSG00000100985 | Matrix metallopeptidase 9 |
| MPO | 4353 | ENSG00000005381 | Myeloperoxidase |
| MTOR | 2475 | ENSG00000198793 | Mechanistic target of rapamycin kinase |
| PADI4 | 23569 | ENSG00000159339 | Peptidyl arginine deiminase 4 |
| PTAFR | 5724 | ENSG00000169403 | Platelet activation factor receptor |
| PIK3CA | 5290 | ENSG00000121879 | Phosphatidylinositol-4,5-bisphosphate 3-kinase |
| RIPK1 | 8737 | ENSG00000137275 | Receptor interacting serine/threonine kinase 1 |
| RIPK3 | 11035 | ENSG00000129465 | Receptor interacting serine/threonine kinase 3 |
| SELP | 6403 | ENSG00000174175 | P-selectin |
| SELPLG | 6404 | ENSG00000110876 | P-selectin receptor |
| SIGLEC14 | 10049587 | ENSG00000254415 | Sialic acid binding Ig like lectin 4 |
| TLR2 | 7097 | ENSG00000137462 | Toll like receptor 2 |
| TLR4 | 7099 | ENSG00000136869 | Toll like receptor 4 |
| TLR7 | 51284 | ENSG00000196664 | Toll like receptor 7 |
| TLR8 | 51311 | ENSG00000101916 | Toll like receptor 8 |
| TNF | 7124 | ENSG00000232810 | Tumor necrosis factor-alpha |

**Supplementary table 2. The differentially expressed Neutrophil extracellular traps-related genes in LUAD.**

| Gene | conMean | treatMean | logFC | pValue |
| --- | --- | --- | --- | --- |
| ALPL | 307.792877966102 | 132.213021706865 | -1.21909557408004 | 2.448957159338e-19 |
| BST1 | 16.1485627118644 | 7.19392096474954 | -1.16655555021654 | 2.33222190512735e-26 |
| CD93 | 208.386071186441 | 45.7783807050093 | -2.18652051190175 | 3.17311967504375e-32 |
| CEACAM3 | 1.50658644067797 | 0.812143970315399 | -0.891476045699676 | 7.17093230630056e-16 |
| CREB5 | 4.52594576271186 | 3.23633506493506 | -0.483858315063225 | 2.25679068204048e-09 |
| CRISPLD2 | 37.8926644067797 | 23.8924098330241 | -0.665366211862894 | 0.000697169465706403 |
| CSF3R | 52.2830661016949 | 24.7147705009276 | -1.08097023958939 | 1.18373203145434e-17 |
| DYSF | 27.2108084745763 | 16.2719337662338 | -0.74179410996112 | 2.31708475280568e-12 |
| FCAR | 1.72722711864407 | 0.823443228200371 | -1.06871670719885 | 1.11872407594471e-07 |
| FCGR3B | 15.8947016949153 | 3.12309833024119 | -2.34749604263135 | 2.34506774298622e-19 |
| CPPED1 | 20.5111406779661 | 13.2484825602968 | -0.630580598712101 | 4.47484780982827e-17 |
| FPR1 | 66.6717 | 18.176393877551 | -1.8750085104239 | 5.2267886199278e-23 |
| FPR2 | 22.1332423728814 | 2.97220408163265 | -2.89660972711982 | 1.30811909191794e-30 |
| G0S2 | 102.723240677966 | 124.927271985158 | 0.282325833887006 | 0.000451221692957957 |
| HPSE | 6.12307288135593 | 8.86078701298701 | 0.53317898907975 | 0.00239183931989573 |
| CXCR1 | 9.18419491525424 | 1.23529294990724 | -2.89430004278259 | 1.7065574223967e-24 |
| CXCR2 | 10.8472406779661 | 1.80555435992579 | -2.5868143350957 | 6.38275391119316e-29 |
| KCNJ15 | 45.0306355932203 | 17.7656968460111 | -1.34181256260907 | 4.56066225208435e-21 |
| LILRB2 | 18.6305745762712 | 11.4371298701299 | -0.703947112935476 | 2.48880133445852e-13 |
| MGAM | 1.00045762711864 | 0.457558070500928 | -1.12863330710246 | 5.36857994807131e-10 |
| MME | 32.3511440677966 | 6.05440371057514 | -2.41775994958338 | 6.67903282528962e-32 |
| PDE4B | 15.4592101694915 | 8.62320148423006 | -0.842171117435885 | 1.17549744053446e-13 |
| S100A12 | 19.8064305084746 | 4.79114508348794 | -2.04752649651269 | 2.42180219949213e-20 |
| SIGLEC5 | 0.449796610169492 | 0.149714842300557 | -1.58705553534366 | 2.05902226648435e-16 |
| SLC22A4 | 8.65646440677966 | 3.7673319109462 | -1.20023475597443 | 7.30760287193359e-09 |
| SLC25A37 | 19.4304661016949 | 33.5191571428571 | 0.786665363174769 | 1.86284801210243e-06 |
| TECPR2 | 10.5859796610169 | 9.28213654916512 | -0.189625961009107 | 0.000235793504481484 |
| VNN3 | 1.73206101694915 | 0.989139146567718 | -0.808244363490774 | 1.09947408351654e-07 |
| AKT1 | 38.493913559322 | 35.8508959183673 | -0.102621180522977 | 0.00118252679095348 |
| AKT2 | 19.3497203389831 | 22.7122675324675 | 0.231159032790891 | 0.0218936452206101 |
| ATG7 | 12.8129423728814 | 10.6634703153989 | -0.264924790611294 | 8.26015602315867e-09 |
| CLEC6A | 2.54766610169492 | 0.813356215213358 | -1.64721697520213 | 7.13560464788549e-16 |
| CSF3 | 102.836894915254 | 4.05935435992579 | -4.66296385950189 | 1.52860544386343e-19 |
| CTSG | 12.3211491525424 | 3.36997087198516 | -1.87032879088947 | 3.4030323310874e-15 |
| CYBB | 218.495401694915 | 92.7334358070501 | -1.2364414046815 | 3.16311514521382e-20 |
| DNASE1 | 2.03864745762712 | 4.55619257884972 | 1.1602164151113 | 1.21145398128641e-15 |
| ELANE | 1.58413728813559 | 1.75798460111317 | 0.150225062417466 | 1.86917846430559e-11 |
| ENTPD4 | 19.3809745762712 | 29.1077315398887 | 0.5867612911364 | 5.21849830174423e-09 |
| F3 | 197.792345762712 | 168.000155658627 | -0.23552402754333 | 6.2653326964078e-07 |
| HMGB1 | 88.3509 | 75.6266890538033 | -0.224349372160865 | 4.1092212826878e-09 |
| IL17A | 0.0276389830508475 | 0.119405936920223 | 2.11109813060003 | 0.000166569867271968 |
| IL1B | 19.9461322033898 | 10.143138961039 | -0.975604830399107 | 1.30589983493632e-14 |
| IL6 | 75.7546338983051 | 10.5208215213358 | -2.8480867784189 | 2.11128697099123e-15 |
| IRAK4 | 12.2526847457627 | 14.7978254174397 | 0.272287283446235 | 0.000715560384877886 |
| ITGAM | 36.9601525423729 | 20.4556786641929 | -0.853469308327531 | 8.54988928394845e-13 |
| ITGB2 | 149.759979661017 | 99.6123244897959 | -0.588255988860213 | 6.2793445146427e-08 |
| KCNN3 | 4.50047966101695 | 2.11583914656772 | -1.08884881910185 | 1.50207889235859e-13 |
| MAPK1 | 89.0141644067797 | 78.3593209647495 | -0.183930027891531 | 4.2838101655137e-06 |
| MAPK3 | 88.3195186440678 | 65.5068348794063 | -0.431086866192454 | 3.36180814691204e-11 |
| MMP9 | 21.9138576271186 | 88.207306122449 | 2.00905468481018 | 2.26508387235649e-14 |
| MPO | 0.764079661016949 | 0.533029499072356 | -0.519507680606867 | 4.08936629014023e-09 |
| MTOR | 15.5313152542373 | 20.3928189239332 | 0.392881206496495 | 5.72990875770843e-05 |
| PADI4 | 1.87932372881356 | 0.332701484230056 | -2.49791339671027 | 1.38365235430229e-22 |
| PTAFR | 56.8571813559322 | 30.051012987013 | -0.919928957028688 | 2.97969662672649e-17 |
| PIK3CA | 6.60843389830509 | 5.96026382189239 | -0.148932223947395 | 1.33527744459441e-05 |
| RIPK1 | 18.5140491525424 | 16.7118146567718 | -0.147752059928752 | 0.0018979629163133 |
| SELP | 36.6562966101695 | 7.27321131725417 | -2.33339663924023 | 1.85019794632387e-32 |
| SELPLG | 122.921845762712 | 45.0640051948052 | -1.44769388723148 | 9.65393810147654e-31 |
| SIGLEC14 | 16.9446525423729 | 6.83998070500928 | -1.30876589351025 | 1.9865890404221e-20 |
| TLR2 | 42.3430101694915 | 30.6542632653061 | -0.466036102553369 | 8.09512490212043e-11 |
| TLR4 | 17.3710169491525 | 5.9878573283859 | -1.53657046394629 | 4.26831835327222e-29 |
| TLR7 | 8.38185254237288 | 4.72996326530612 | -0.825440162009126 | 9.44448919467907e-15 |
| TLR8 | 20.0600762711864 | 6.15913450834879 | -1.70352755087723 | 2.35453528564838e-27 |
| TNF | 5.78846271186441 | 4.53080909090909 | -0.353411547995318 | 1.59349198363535e-05 |
